# Phosphodiesterase-4 Inhibitors for Non-COPD Respiratory Diseases

**DOI:** 10.3389/fphar.2021.518345

**Published:** 2021-08-05

**Authors:** Theerasuk Kawamatawong

**Affiliations:** Division of Pulmonary and Critical Care Medicine, Department of Medicine, Ramathibodi Hospital, Mahidol University, Bangkok, Thailand

**Keywords:** roflumilast, chronic cough, bronchiectases, asthma, phosphodiesterase

## Abstract

Selective phosphodiesterase (PDE) inhibitors are a class of nonsteroid anti-inflammatory drugs for treating chronic inflammatory diseases. Modulation of systemic and airway inflammation is their pivotal mechanism of action. Furthermore, PDE inhibitors modulate cough reflex and inhibit airway mucus secretion. Roflumilast, a selective PDE4 inhibitor, has been extensively studied for the efficacy and safety in chronic obstructive pulmonary disease (COPD) patients. According to the mechanisms of action, the potential roles of PDE inhibitors in treating chronic respiratory diseases including severe asthma, asthma-COPD overlap (ACO), noncystic fibrosis bronchiectasis, and chronic cough are discussed. Since roflumilast inhibits airway eosinophilia and neutrophilia in COPD patients, it reduces COPD exacerbations in the presence of chronic bronchitis in addition to baseline therapies. The clinical studies in asthma patients have shown the comparable efficacy of roflumilast to inhaled corticosteroids for improving lung function. However, the clinical trials of roflumilast in severe asthma have been limited. Although ACO is common and is also associated with poor outcomes, there is no clinical trial regarding its efficacy in patients with ACO despite a promising role in reducing COPD exacerbation. Since mucus hypersecretion is a result of neutrophil secretagogue in patients with chronic bronchitis, experimental studies have shown that PDE4s are regulators of the cystic fibrosis transmembrane conductance regulator (CFTR) in human airway epithelial cells. Besides, goblet cell hyperplasia is associated with an increased expression of PDE. Bronchiectasis and chronic bronchitis are considered neutrophilic airway diseases presenting with mucus hypersecretion. They commonly coexist and thus lead to severe disease. The role of roflumilast in noncystic fibrosis bronchiectasis is under investigation in clinical trials. Lastly, PDE inhibitors have been shown modulating cough from bronchodilation, suppressing transient receptors potential (TRP), and anti-inflammatory properties. Hence, there is the potential role of the drug in the management of unexplained cough. However, clinical trials for examining its antitussive efficacy are pivotal. In conclusion, selective PDE4 inhibitors may be potential treatment options for chronic respiratory diseases apart from COPD due to their promising mechanisms of action.

## Introduction

Phosphodiesterase (PDE) is an enzyme involved in the pathogenesis of chronic inflammatory diseases and degenerative diseases, for instance, asthma, chronic obstructive pulmonary disease (COPD), psoriatic arthritis, atopic dermatitis, and dementia of Alzheimer. PDE hydrolyzes substrates, nucleotide cyclic adenosine monophosphate (cAMP), and cyclic guanosine monophosphate (cGMP) to inactive metabolites as adenosine-5-monophosphate (5-AMP) and guanosine-5-monophosphate (5-GMP), respectively (Rabe, 2011). The inhibition of PDE4 produces diverse clinical effects via the elevation in the intracellular cAMP level. Therefore, subsequent gene activation and protein transcription involved in the pathogenesis of chronic inflammatory diseases are regulated. The selective PDE4 inhibitors have been extensively studied for being the novel anti-inflammatory therapies in various diseases (Li et al., 2018). The prototypic drug is roflumilast; the oral selective PDE4 inhibitor has been approved for maintenance therapy for severe COPD with chronic bronchitis (Kawamatawong, 2017). Furthermore, apremilast has been approved in the treatment of active psoriatic arthritis and moderate-to-severe plaque psoriasis (Li et al., 2018). Last, but not least, crisaborole has been approved for the topical treatment of atopic dermatitis (Lazaros et al., 2017). Despite the robust mechanisms of action of PDE4 inhibitors, roflumilast and other selective PDE4 inhibitors have been investigated in preclinical and clinical studies for the treatment of other chronic respiratory diseases, metabolic diseases, dermatologic diseases, and neurological degenerative diseases. This article will discuss the evidence of roflumilast and other PDE4 inhibitors for treating chronic inflammatory airway disease beyond COPD including specific COPD phenotypes with comorbidities, severe asthma, noncystic fibrosis bronchiectasis, chronic rhinosinusitis, and treatment of chronic cough.

## Basic Pharmacology of Phosphodiesterase-4 (PDE4) Inhibitors

cAMP is a key secondary messenger for intracellular signaling processes (Francis and Corbin, 1999). cAMP produces the functional effect on the protein kinase A (PKA) pathway by phosphorylating the protein causing activation. cAMP exerts an effect beyond the PKA pathway by activating the exchange protein activated by cAMP (EPAC) resulting in a change in protein functions and cellular shape change (Grandoch et al., 2010). The classification of PDEs is according to their binding affinity of PDE inhibitors to a different substrate and their cellular and tissue distribution. PDE4 is cAMP-selective PDE and is abundant in inflammatory cells, airway cells, and lung tissue. PDE4 is classified as PDE4A, PDE4B, PDE4C, and PDE4D according to their tissue distribution and genetic encoding (Halpin, 2008). Rolipram, the first PDE4 inhibitor, was developed for the treatment of psychotic diseases but severe treatment-related side effects limited the clinical utility. The second-generation PDE4 inhibitors including cilomilast and roflumilast were later developed. The most extensively studied PDE4 inhibitor in respiratory diseases is roflumilast (Page and Spina, 2012). Roflumilast and its metabolite roflumilast-N-oxide have the potent inhibitory effect on the PDE4 enzyme compared to nonselective PDE inhibitors such as theophylline (Boswell-Smith et al., 2006; Hatzelmann et al., 2010). The mechanisms of roflumilast and roflumilast-N-oxide are exhibited in both inflammatory cells and structural cells involved in the pathogenesis of chronic respiratory diseases. The inhibitory effect of these drugs on various inflammatory cell functions has been shown in experimental models. Roflumilast inhibits human lung macrophages release of inflammatory cytokines including CC-and CXC motif chemokines and TNF-α (Buenestado et al., 2013). Rolipram, a PDE4 inhibitor, suppresses the IgE-dependent generation of IL-4, IL-13 and histamine from basophils (Eskandari et al., 2004). Roflumilast potently inhibits fMLP- and C5a-induced eosinophil reactive oxygen species (ROS) formation (Hatzelmann and Schudt, 2001). Moreover, roflumilast inhibits neutrophils’ function by suppressing neutrophilic releases of their mediators, for instance, leukotriene B4 (LTB4), neutrophils elastases (NEs), matrix metalloproteinase-9 (MMP-9), and chemoattractant-stimulated neutrophil migration (CXCL1) (Hatzelmann et al., 2010). The roflumilast-N-oxide metabolite in combination with formoterol, a long-acting β2 agonist, enhances the effect of dexamethasone on airway smooth muscle cells (Patel et al., 2017). The inhibitory effect on the contractile activity of airway smooth muscle cells via mitogen-activated protein kinase phosphatase 1 (MKP-1) results in a synergistic bronchodilator effect (Patel et al., 2015). Roflumilast also inhibits the profibrotic growth factor (TGF-β) and attenuates chemotaxis of fibroblasts that underlie the pathogenesis of airways and parenchymal lung fibrosis (Kohyama et al., 2002; Togo et al., 2009; Udalov et al., 2010). Roflumilast inhibits bronchial epithelial cell release of TNF-α. Also, roflumilast decreases the expression of MUC5AC expression in human airway epithelial cells induced by epidermal growth factor (EGF) (Mata et al., 2005). Hence, it exerts the potential treatment effect on mucus hypersecretion (Takeyama et al., 2001). Furthermore, roflumilast activates the cystic fibrosis transmembrane conductance regulator (CFTR) in airway epithelium cells (Liu et al., 2005). Roflumilast-N-oxide also improves cilia motility of cigarette smoke–injured ciliated human bronchial epithelium (Milara et al., 2012). Last, but not least, roflumilast can increase airway-surface liquid (ASL) hydration in cigarette smoke–exposed human bronchial epithelial cultures (HBECs), which is beneficial for mucus dehydration (Tyrrell et al., 2015). These potential mechanisms of roflumilast are for promoting mucus clearance in COPD with chronic bronchitis and other suppurative airway diseases.

## Anti-Inflammatory Effects of Roflumilast on COPD

The anti-inflammatory effects of roflumilast have been clinically investigated in patients with COPD and asthma. The attenuation of airway inflammation in COPD patients taking oral roflumilast has demonstrated the reduction in sputum cell counts including neutrophils, eosinophils, and macrophages. It also decreased mediators from those inflammatory cells, for instance, LTB4 and NE (Grootendorst et al., 2007). Roflumilast reduces sputum proline–glycine–proline (PGP), a neutrophil-degrading tissue collagen product, and inhibits neutrophilic inflammation in COPD patients. Despite that roflumilast decreased PGP and prolyl endopeptidase, it lacks an effect on leukotriene A4 hydrolase activity (Wells et al., 2015). This finding emphasized the roles of roflumilast on the neutrophilic airway inflammation in COPD. The bronchial biopsy study of COPD patients who were treated with roflumilast has shown the reduction of eosinophilic infiltration in bronchial tissue compared to placebo without the effect on blood eosinophilia. However, it failed to reduce CD8^+^ cell infiltration from the bronchial biopsy (Rabe et al., 2018). These findings emphasize the benefit of roflumilast on COPD with eosinophilic airway inflammation. Bronchial biopsies of exacerbated COPD patients reveal the marked increase in airway eosinophils and moderately increased airway neutrophils compared with those of stable COPD (Saetta et al., 1994). The protective roles of CD8 lymphocytes in respiratory viral infection have been noted (Gilchuk et al., 2016). Nevertheless, the role of roflumilast for protection against viral infection in asthma and COPD is not clear. The higher prevalence of antibiotic-treated exacerbation in COPD receiving inhaled corticosteroid (ICS)/LABA has been shown in the INSPIRE study. The LAMA-treated COPD has been shown the increased in more prevalence COPD exacerbation requiring systemic corticosteroid (Wedzicha et al., 2008). The meta-analysis emphasizes the immunosuppressive effect of ICS-containing regimens and the risk of pneumonia in COPD patients (Yang et al., 2019). Roflumilast reduces the rate of moderate or severe exacerbations or antibiotic-treated exacerbations in COPD patients with a history of more than three exacerbations and/or one or more hospitalizations in the prior year (Martinez et al., 2016). There was no conclusive evidence of roflumilast increasing the risk of viral exacerbation in COPD. However, ICS/LABA/LAMA-treated COPD gains the benefit for preventing exacerbation regardless of causes. The putative mechanism of roflumilast on COPD exacerbation may be related to modulated underlying airway inflammation in COPD. Moreover, roflumilast inhibits neutrophil chemotaxis directly via a cAMP-mediated mechanism requiring activation of Epac1. Hence, the drug reduces neutrophilic inflammation in COPD patients (Dunne et al., 2019). The differential effects of roflumilast on airway inflammation in patients with the different COPD phenotypes lead to treatable traits of airway diseases such as asthma with neutrophilic inflammation and COPD with eosinophilic inflammation. To explore the clinical efficacy, the well-designed randomized clinical studies are required for unmet clinical needs regarding the burden of chronic inflammatory airway diseases.

## Roflumilast as COPD Maintenance Therapy

PDE4 inhibitors have been investigated for their efficacy in the treatment of COPD for 2 decades. Roflumilast and cilomilast were developed in the initial phase. Roflumilast is the only PDE4 inhibitor that was approved by the United States FDA and EMA for maintenance therapy of COPD. Roflumilast provides clinical benefit in terms of improving patient lung function and reducing the risk of COPD exacerbation with an impact on the quality of life of COPD patients with frequent exacerbations and persistent symptoms despite maximal therapy (Rabe et al., 2005; Calverley et al., 2007; Calverley et al., 2009; Fabbri et al., 2009; Rennard et al., 2011; Zheng et al., 2014; Martinez et al., 2015; Martinez et al., 2016; Chong et al., 2017; Shen et al., 2018). Roflumilast is the only oral PDE4 inhibitor that has been extensively investigated for its efficacy in asthma and COPD. The improvement of lung function is key for being approved as add-on therapy in asthma and COPD.

The physiological basis of roflumilast beyond its anti-inflammatory properties has been investigated. Roflumilast improves airway function including direct bronchodilator activity, improving mucociliary function in the cigarette smoke exposure model (Milara et al., 2012), and preventing airway fibrosis related to stem cell factor (SFC) inhibition (Kim et al., 2016). The add-on roflumilast to inhaled tiotropium bromide (LAMA) or inhaled salmeterol (LABA) exhibits lung function improvement in COPD patients ranging from 49 to 80 ml compared to placebo (Rabe, 2011). However, the side effects of roflumilast including emesis and diarrhea are commonly observed. The common side effects of roflumilast that were observed from pool analysis are diarrhea (9.5%), weight loss (7.5%), nausea (4.7%), back pain (3.2%), influenza (2.8%), insomnia (2.4%), and decreased appetite (2.1%). These side effects are not different from the placebo (Michalski, et al., 2012). A dose of roflumilast 250 µg once daily (OD) for four weeks before dose escalation to an approved maintenance dose of 500 µg OD has been investigated (Watz, et al., 2018). This approach resulted in reduced treatment discontinuation and improved patient tolerability. The fewer treatment discontinuations and lower rates of adverse events are notable compared with the standard dose. The clinical evidence has shown exacerbation reduction and improving lung function from adding roflumilast to COPD standard treatment. The evidence of roflumilast in the treatment of COPD patients in a clinical trial was summarized in Table 1. Despite the benefit of oral administration that a PDE4 inhibitor has demonstrated in the clinical use, the PDE4D inhibition is associated with a pronounced emetic side effect compared to the anti-inflammatory effect related to PDE4B inhibition. Hence, improving the therapeutic index of the selective PDE4B is a potential target for minimizing the side effects. Roflumilast exhibits more selective PDE4B inhibition resulting in a better therapeutic index compared to that of cilomilast that is prominent PDE4D inhibition. There is limited clinical evidence regarding the direct PDE4B inhibition without PDE4 modulation which is a gap in drug development (Fox et al., 2014).

**TABLE 1:** Summary of randomized clinical trials of roflumilast in COPD as COPD maintenance therapy.

Authors, year	Acronym	Baseline patient characteristics	Baseline post-BD FEV_1_	Treatment	Duration (weeks)	Efficacy outcomes
Rabe et al. (2005)	RECORD	AECOPD-NR	30–80% predicted	RF 250 μg and RF 500 μg PBO OD	24	↑ Post-BD FEV_1_ (74 ml in RF 250 µg and 97 ml in RF 500 µg) vs. PBO ↑ HR-QOL in RF
Calverley et al. (2007)	RATIO	AECOPD-NR	≤50% predicted	RF 500 µg PBO OD	52	↑ Post-BD FEV_1_ (39 ml in RF) vs. PBO
Calverley et al. (2009)	AURA (M-124)	AECOPD history and chronic bronchitis	≤50% predicted	RF 500 µg PBO OD	52	↑ Post-BD FEV_1_ (48 ml in RF) vs. PBO
	HERMES (M-125)					Rate of ECOPD 1.14 in RF and 1.37 in PBO
Fabbri et al. (2009)	EOS (M-127)	Moderate-to-severe COPD	40–70% predicted	RF 500 µg PBO OD	24	↑ Pre-BD FEV_1_ (49 ml in RF) in SM-treated COPD ↑ Pre-BD FEV_1_ (80 ml in RF) in TIO-treated COPD
	HELIOS (M-128)					
Zheng et al. (2014)	ACROSS	Asian COPD on ICS/LABA or LAMA	40–70% predicted	RF 500 µg PBO OD	24	↑ Post-BD FEV_1_ (71 ml in RF) vs. PBO
Martinez et al. (2015)	REACT	≥2 AECOPD in the previous year on ICS/LABA		RF 500 µg PBO OD	52	↓ Rate of AECOPD 13.2% (Poisson regression) and 14.2 (negative binomial regression) in RF
Martinez et al. (2016)	RE(2)SPOND	≥2 AECOPD in the previous year and/or hospitalization on LAMA or ICS/LABA for 3 months		RF 500 µg PBO OD	52	↓ Rate of moderate-to-severe AECOPD in patients with ≥3 exacerbations and/or >1 hospitalization in the previous year (post hoc analysis)

AECOPD, acute exacerbation of COPD; Post-BD FEV_1_, postbronchodilator FEV_1_; Pre-BD FEV_1_, prebronchodilator FEV_1_; ICS/LABA, inhaled corticosteriod/long-action β2 agonist; LAMA, long-acting antimuscarinics; RF, roflumilast; PBO, placebo; COPD, chronic obstructive pulmonary diseases; OD, once daily.

Selective PDE4 and dual PDE3/4 inhibitors are potential therapeutic agents for treating chronic respiratory diseases. PDE4 inhibitors exert their anti-inflammatory, while dual PDE3/4 inhibitors exhibit their bifunctional bronchodilator/anti-inflammatory agents. There is robust evidence to suggest that dual PDE3/4 inhibition has an additive or synergistic effect on suppressing inflammatory mediators’ release from other cell types, which also express PDE3 that is thought to play a role in COPD (Abbott-Banner and Page, 2014).

The position and clinical evidence regarding inhibitor targeting other than PDE4 including PDE1 and PDE3 are promising. The PDE1 inhibitors modulate the physiological effect via vasodilatation and preventing cardiac remodeling. They have been investigated as therapeutic agents for neurodegenerative disorders including Alzheimer’s disease and Parkinson’s disease. (Medina, 2011). PDE1 and PDE3 play a protective role in pathological cardiac remodeling and dysfunction by the modulation of cyclic nucleotides such as cardiac failure. The PDE3 inhibitors exhibit the inotropic effect on cardiac failure (Movsesian et al., 2011). The anti-inflammation and bronchodilator therapy have been investigated in PDE3 inhibitors, enoximone, and milrinone (Beute et al., 2018). There is limited evidence of PDE3 inhibitors in asthma and COPD despite that the selectivity of PDE3 for cAMP is 20 times that of PDE4 (Boswell-Smith et al., 2006). Cilostazol is a PDE3 inhibitor, showing its beneficial effect on bronchial hyperresponsiveness in elder asthmatics (Fujimura, et al., 1995). The benefit of treatment with intravenous enoximone (PDE3/4 inhibitor) in status asthmaticus has been shown (Beute, 2014). The putative mechanism is that enoximone is attenuating airway hyperresponsiveness in house dust mite–sensitized mice (Beute et al., 2015). Cilostazol has been shown its clinical benefit and safety in the treatment of intermittent claudication (Dawson et al., 1998). Furthermore, studies have demonstrated the decreased expression of PDE3 and PDE4 in cardiac hypertrophy or heart failure (Leroy et al., 2018). PDE3 inhibitors including milrinone and enoximone have been shown to improve the short-term cardiac functions in heart failure patients. However, the increased adverse events and the lack of mortality benefit of oral milrinone compared to placebo have been established in severe heart failure (Packer et al., 1991). The underlying mechanism is potentially related to PDE3 inhibitor–induced downregulation of PDE3A expression resulting in cardiomyocyte apoptosis (Ding et al., 2005). Likewise, low-dose oral enoximone has been proved safe for patients with severe heart failure. Despite the mortality, the benefit is not different from placebo (Metra et al., 2009). For these reasons, the preclinical and clinical evidence regarding PDE4 inhibitors in obstructive airway diseases is more prominent than other non-PDE4 inhibitors.

The investigational PDE4 inhibitors including oral and inhalation forms have been examined for their efficacy and safety. Inhaled PDE4 inhibitors, for instance, inhaled CHF 6001, have been clinically investigated in asthma and COPD patients (Phillips, 2020). The only PDE4 inhibitors that have been approved in the treatment of dermatologic and respiratory diseases are apremilast, crisaborole, and roflumilast. Roflumilast’s efficacy and safety have been shown for a decade. However, the side effects are a major limitation. Hence, the other formulation of PDE4 inhibitors such as inhaled PDE4 inhibitors is currently being investigated for their effect on COPD and asthma. The investigational oral PDE4 inhibitors cilomilast, ibudilast, oglemilast, and tofimilast are currently being clinically investigated (Beghe et al., 2013).

Subgroup analysis has shown that the benefit of decreased exacerbation is noted in chronic bronchitis with and without pulmonary emphysema. Roflumilast has been positioned in the Global Obstructive Lung Disease (GOLD) document (Global Initiative for Chronic Obstructive Lung Disease (GOLD), 2019) for being add-on therapy or follow-up therapy options for COPD after the initial option in the patients with persistent exacerbations. However, the efficacy of roflumilast on COPD experienced frequent exacerbation (≥2 exacerbations in the past year) and/or hospitalization has been demonstrated in a minority of COPD patients according to these clinical studies. Integrated analysis of M124 and M125 has shown that roflumilast reduced the rate of COPD exacerbations per patient per year by 22.3% compared to placebo in COPD patients who experienced ≥2 exacerbations in the previous years, while the rate of COPD exacerbations was reduced by 16.5% by roflumilast compared to placebo in COPD patients who experienced <2 exacerbations in the previous year (Calverley et al., 2009). Additional clinical studies examined the benefit of roflumilast in frequent exacerbated COPD by including frequent COPD exacerbators (>2 exacerbations in the past years) despite maximized COPD maintenance therapy. Roflumilast further reduced the rate of exacerbation COPD suffered from frequent exacerbations REACT study and RE(2)SPOND study (Martinez et al., 2015; Martinez et al., 2016). The regular ICS treatment prevents the progression of asthma airway remodeling related to frequent exacerbations. However, the effect of ICS on airway remodeling is closely related to the timing of ICS treatment (O’Byrne et al., 2009). ICS preventing airway injuries from repeated exacerbations is the key mechanism (Bai et al., 2007). Despite the fact that bronchodilators are a mainstay for COPD treatment, the ICS-containing regimens for COPD are limited for frequent exacerbated COPD and the presence of blood eosinophilia (Agusti et al., 2018). Hence, roflumilast may fill the treatment gap as nonsteroid anti-inflammatory therapy for high-risk COPD patients (Singh et al., 2016b). To date, the ICS’s sparing effect of roflumilast or PDE4 inhibitors on COPD has never been shown in well-designed clinical studies. Despite the benefit in terms of future risk reduction, roflumilast has shown the clinical benefit of reducing fat-free mass and reducing the level of glycosylated hemoglobin in patients with COPD. According to the mechanisms of action, roflumilast may be beneficial in COPD comorbidities and extrapulmonary features including chronic rhinosinusitis, COPD overlap with obstructive sleep apnea, COPD with skeletal muscle dysfunction, and COPD with metabolic syndrome including diabetes.

## Roles of Roflumilast in COPD Patients With Specific Phenotypes and Comorbidities

**FIGURE 1:**
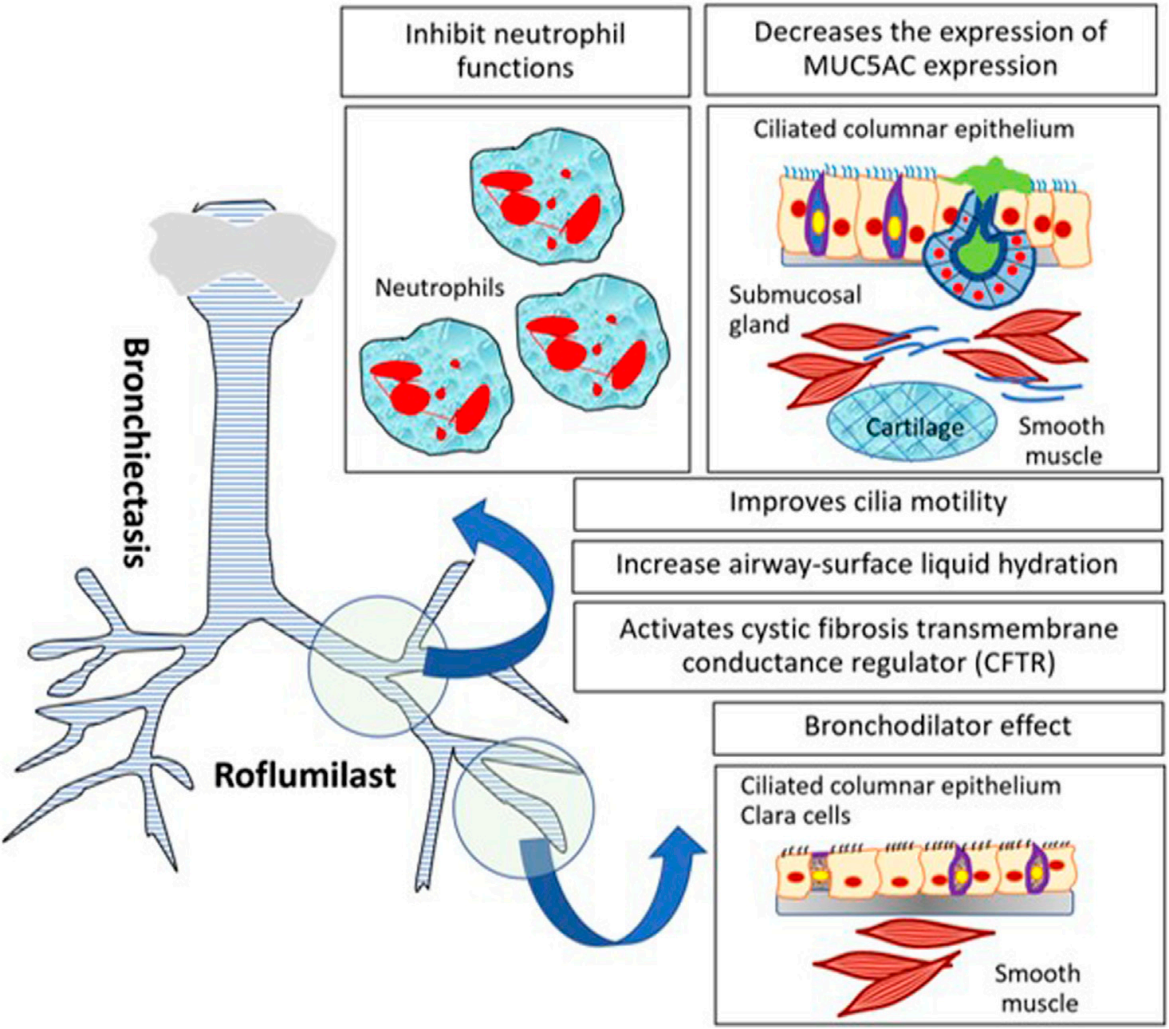
The potential role of roflumilast and PDE4 inhibitors in COPD with specific phenotypes or COPD with comorbidities.

Roflumilast has been investigated for clinical effects on COPD patients with specific phenotypes such as COPD with frequent exacerbations and chronic bronchitis despite maximized treatment and specific comorbidities, for instance, effect on obesity, metabolic diseases, and cardiovascular diseases. This drug also has a potential mechanism in other conditions associated with COPD, for instance, COPD with small airway diseases and pulmonary hypertension. Roles of roflumilast and PDE4 inhibitors in these COPD patients are shown in Figure 1.

## COPD With Superexacerbations

The efficacy of roflumilast for reducing exacerbation was demonstrated in controlled studies (Rennard et al. 2011; Chong et al., 2017; Shen et al., 2018). This benefit is favorable in COPD with chronic bronchitis phenotypes in the presence and absence of pulmonary emphysema (Rennard et al., 2011). Since the increasing awareness regarding the use of ICS in COPD with a high risk of exacerbation is addressed, ICS-containing regimens, for instance, ICS with the long-acting β2 agonists, are recommended for COPD patients with frequent exacerbations having blood eosinophilia or the presence of coexisting asthma (Agusti et al., 2018). The risk of pneumonia in the long-term use of ICS in COPD must be taken into account. The attempt to ICS step-down has been done in well-designed studies in patients with COPD to a background of dual bronchodilator therapy (Magnussen et al., 2014; Chapman et al., 2018). Hence, other nonsteroidal anti-inflammatory agents are considered in COPD with persistent exacerbation despite the bronchodilator maximization. The benefit of adding on roflumilast to either monotherapy of long-acting antimuscarinic (LAMA) or long-acting β2 agonist (LABA) has shown FEV_1_ improvement. Also, the add-on benefit of roflumilast to combination therapies, for instance, ICS/LABA or ICS/LABA/LAMA, has been investigated systematically. The efficacy of therapies for reducing exacerbations was a major concern in COPD with superexacerbations (≥3 times a year) (Wedzicha et al., 2013). Since most of the clinical studies examining the efficacy of COPD therapies have included ≥1 exacerbations in the past year, some of the recruited patients were frequent exacerbators (>2 exacerbations in the past year). For instance, one-fifth of the patients experiencing ≥2 COPD exacerbations in the past year were recruited in a study examining the efficacy of glycopyrronium bromide/indacaterol for preventing COPD exacerbation compared to that of salmeterol/fluticasone propionate (FLAME) (Wedzicha et al., 2016). Besides, COPD patients with at least one moderate or severe exacerbation requiring systemic glucocorticoids and/or antibiotics or hospitalization in the previous year were recruited in a study examining the effect of tiotropium bromide/olodaterol compared to that of tiotropium bromide in patients with moderate-to-very severe COPD (DYNAGITO) (Calverley et al., 2018). Furthermore, half of the COPD patients who experienced ≥2 moderate-to-severe exacerbations in the past year were recruited in studies examining the efficacy of fluticasone furoate/vilanterol/umeclidinium bromide compared to that of their dual bronchodilator and ICS/LABA counterpart (IMPACT) (Lipson et al., 2018). As previously mentioned, roflumilast exhibits the roles in the escalation therapy paradigm for being add-on oral agents to inhaled combination treatments, for instance, LAMA/LABA, ICS/LABA, and ICS/LABA/LAMA, in superexacerbated COPD for preventing or reducing exacerbations.

## COPD With Obesity

Body mass index (BMI) is considered the prognostic marker of COPD mortality (Celli et al., 2004). Sarcopenia is associated with low appetite and increased systemic inflammation in COPD (Byun et al., 2017). Obese COPD is not uncommon in the Western world (García-Rio et al., 2014). The comorbidities are more frequent in obese COPD than in normal weight and underweight COPD (Knorst et al., 2011). The energy restriction and resistance exercise training improve 6-minute walk distance and SGRQ by preservation of fat-free mass (FFM) (McDonald et al., 2014). Furthermore, weight reduction intervention also improves cardiometabolic markers in obese COPD (Negewo et al., 2016). Since roflumilast has been shown the metabolic effect by reducing body weight in terms of fat mass. The magnitude of weight reduction in roflumilast is minimal for clinical improvement. Besides, the reduced glycosylated hemoglobin has been shown in the single study of roflumilast-treated COPD patients (Wouters et al., 2012). However, the efficacy of roflumilast on metabolic diseases including diabetes in patients with obese COPD needs further investigation.

## COPD With Small Airway Diseases

Small airway fibrosis is one pathophysiological change in COPD apart from chronic mucus hypersecretion and pulmonary emphysema (Barnes, 2000). Small airway dysfunction (SAD) and preceding pulmonary emphysema contributed to lung hyperinflation and symptoms of COPD (McDonough et al., 2011; Pisi et al., 2015). An experimental study examined the role of PDE inhibition in small airway fibroblasts. The small particle drug, for instance, extrafine particle ICS/LABA or the novel technology of an inhaler, is crucial for reaching SAD. SAD was also associated with the FVC or volume responsiveness after bronchodilator administration in COPD (Vos et al., 2016). Roflumilast, as an oral therapeutic agent, may improve small airway function in COPD. Roflumilast reduced regional hyperinflation examined by functional resonance imaging (FRI) in COPD previously undertreated by inhaled triple therapies (ICS/LABA/LAMA) (De Backer et al., 2014). SAD is prevalent in asthma and COPD. According to the epidemiological studies, SAD is described in all spectra of asthma which have been shown in the Assessment of Small Airways Involvement in Asthma (ATLANTIS study) (Postma et al., 2019). The oral leukotriene modifier, montelukast, improves regional air trapping as a marker of small airway function in ICS-naïve asthma (Zeidler et al., 2006). The effect of roflumilast on SAD was shown in FRI as mentioned previously. Hence, the role of inhaled formulation of a selective PDE4 inhibitor in modulating SAD in asthma and COPD is promising. To date, the long-term clinical studies examining the role of roflumilast on small airway functions and clinical outcomes in COPD need to be investigated.

## COPD With Coronary Artery Diseases

Cardiovascular disease is a common cause of mortality in COPD patients. The more symptomatic COPD may underly by the occult cardiovascular diseases and low-grade systemic inflammation. A previous study has shown that roflumilast treatment is safe in terms of cardiovascular side effects (White et al., 2013). Apart from the cardiovascular side effect, the potential benefit of roflumilast on subclinical atherosclerosis in COPD has been investigated. Despite improvement in 6-minute walk distance, roflumilast fails to improve the arterial stiffness and evidences endothelial dysfunction when compared to placebo (Urban et al., 2017). However, the benefit of roflumilast regarding the protective effect on coronary artery diseases in COPD has to be investigated.

## COPD With Obstructive Sleep Apnea

The increase in body fat in obesity is associated with the increase in tongue fat contributing to upper airway obstruction, which is associated with obstructive sleep apnea (OSA). The prevalence of OSA in COPD is varied (Weitzenblum et al., 2008). Besides, the COPD OSA overlap syndrome is associated with an increased risk of poor outcomes including death and exacerbation requiring hospitalization (Marin et al., 2010). The positive airway pressure (PAP) treatment was associated with improved survival and decreased hospitalizations in patients with overlap syndrome (Hiestand and Phillips, 2008). The previous studies have shown that surgical weight reduction inconsistently improves BMI and AHI in OSA (Mitchell et al., 2014; Wong et al., 2018). There is insufficient evidence to recommend the use of pharmacologic therapy, for instance, donepezil and fluticasone in the treatment of OSA (Mason et al., 2013). However, the benefit of roflumilast as being the drug for treating COPD OSA overlap has never been investigated. Extrapolating the benefit of roflumilast in weight loss is minimal (reduced BMI of 2.4 kg/m^2^) and may not convey the clinical benefit on OSAH in obese COPD patients with OSA.

## COPD Patients With Pulmonary Hypertension

Chronic hypoxemia is a key driver for the development of pulmonary hypertension (PH) associated with chronic lung diseases. Prevalence of COPD associated with PH is not uncommon and varied according to the degree of airflow obstruction, the definition of PH, and the method of diagnostic assessment (Naeije, 2005; Gologanu et al., 2013; Nathan et al., 2018). The magnitude of PH is underestimated by echocardiography due to pulmonary hyperinflation (Laaban et al., 1989). Since oxygen therapy is the key therapeutic for hypoxemia in COPD, the effects on pulmonary hemodynamics and exercise capacity of pulmonary arterial hypertension (PAH)-specific drugs including sildenafil and bosentan in COPD with PH were shown in clinical studies (Valerio et al., 2009; Vitulo et al., 2017). However, the efficacy of the PAH-specific drug in the treatment of COPD-related PH is limited and heterogenous (Jyothula and Safdar, 2009). To date, clinical trials are needed before the use of selective PDE4 inhibitors including roflumilast can be recommended for PH-associated COPD.

## COPD and Chronic Rhinosinusitis

The current evidence of roflumilast’s effect on chronic sinusitis has been investigated. The therapeutic roles of roflumilast in allergic rhinitis were examined for decades (Schmidt et al., 2001). Roflumilast rapidly improves nasal symptoms of AR compared to placebo. However, the limitation of roflumilast is systemic side effects compared to nasal corticosteroid. Hence, there are no further clinical trials of roflumilast and other oral PDE4 inhibitors in allergic rhinitis (Janosova et al., 2020).

The inhaled formulation of a PDE4 inhibitor has been investigated in patients with asthma and COPD. The aims are to improve their tolerability and maximize therapeutic effect (Phillips, 2020). Inhaled CHF6001 is delivered in a dry-powder inhaler (DPI). The allergen-induced late asthmatic response (LAR) in asthmatic patients is attenuated by inhaled CHF6001 (Singh et al., 2016a). In addition, the decreased inflammatory biomarkers in the sputum in CHF 6001 decreases the inflammatory biomarkers in the sputum in COPD patients (Singh et al., 2019). However, the intranasal formulation of a PDE inhibitor has been investigated. Intranasal theophylline methyl propylparaben treatment is safer and more effective in improving hyposmia and hypogeusia than oral theophylline anhydrous in rhinitis (Henkin et al., 2012). These findings indicate that a selective PDE4 inhibitor and an intranasal PDE4 inhibitor may improve subjective and objective measurements of smell function. The potential role of PDE4 inhibitors in allergic rhinitis is related to the differential PDE4D gene expression in patients with allergic rhinitis and nasal polyposis (Apuhan et al., 2013). Hence, the new formulation, for instance, nasal spray, may be the potential solution for managing chronic rhinosinusitis (Janosova et al., 2020).

Asthma coexisting with upper airway disease has been emphasized as united airway disease (Giavina-Bianchi et al., 2016). Rhinosinusitis is frequently associated with asthma and COPD. The impaired nasal function in rhinosinusitis is closed related to bronchial pathology (Hellings et al., 2010). Chronic rhinosinusitis is the common comorbidity in asthma apart from allergic rhinitis. In addition, chronic rhinosinusitis has been reported in COPD and affecting the patient’s quality of life. The nasal inflammation mimics that of the bronchi in COPD patients (Håkansson et al., 2013). Hence, there is a potential role of the PDE4 inhibitor in asthma and COPD patients facing complication with upper airway disease including allergic rhinitis and chronic rhinosinusitis. The treatment using a single approach focusing on both upper airway and lower airways pathology related to type 2 inflammation including oral leukotriene modifier, type 2 biologic agents, and specific immunotherapy has been positioned for a decade (Bousquet et al., 2008). Furthermore, the non–type 2 inflammatory target, low-dose macrolide has been investigated for its role in both upper and lower airway diseases, for instance, asthma, COPD, chronic rhinosinusitis, and diffuse panbronchiolitis (Asano et al., 2012).

The effect of selective PDE4 inhibitors on mucociliary activity in the upper and lower airways has been studied in an *in vitro* model. The effects of rolipram (specific PDE4 inhibitor), milrinone (specific PDE3 inhibitor), and zaprinast (specific PDE5 inhibitor) were investigated in rabbit maxillary sinus and trachea. The different selective PDE inhibitors exhibited the differential effect on ciliary beat frequency (CBF) in rabbit sinus and trachea (Cervin and Lindgrenet, 1998).

The novel oral PDE4 inhibitor, ibudilast, has been investigated for treatment of airway mucus hypersecretion and postnasal drip syndrome (PNDS) in patients with chronic inflammatory airway disease coexisting with chronic sinusitis. The 8-week treatment of ibudilast improves sputum and postnasal drip symptoms regardless of the previous macrolide and corticosteroid treatment. The putative mechanisms are reducing glycoprotein secretion and stimulating bronchial ciliary movement (Tagaya et al., 2010).

The selective PDE1 or PDE4 inhibitor or dual PDE inhibitors (PDE3/4) raise the intracellular level of cyclic nucleotides in airway epithelial cells. Consequently, they may be the potential target in the development of new inhaled mucokinetic drugs. Further studies are needed for examining the cilia-modulating properties of PDE inhibitors (Joskova et al., 2020).

## PDE4 Inhibitors in Asthma Treatment

The increased bronchial hyperresponsiveness (BHR) is a hallmark of asthma pathophysiology. The modulation of BHR is the target of asthmatic treatment. The prevention of allergen-induced early- and late-phase asthmatic reactions (EAR and LAR) is the hallmark of antiasthmatic drug efficacy, for instance, ICS and roflumilast. Previous studies have shown that roflumilast attenuates early- and late-phase asthmatic reaction after allergen challenge in different fashions. Single-dose orally administered roflumilast reduced the decrease in FEV_1_ from allergen challenge compared with that of placebo after 2 h allergen challenge. However, early asthmatic responses to allergen challenge were not significantly reduced by the single dose of roflumilast (Louw et al., 2007). Further, the longer duration of treatment of roflumilast in asthma (7–10 days) has shown the modulation effect on early asthmatic reaction (Van Schalkwyk et al., 2005). Also, roflumilast administered for 2 weeks has been shown to suppress allergen-induced LAR including the influx of inflammatory cells such as eosinophils, basophils, and neutrophils to the airways (Gauvreau et al., 2011). However, roflumilast does not affect early-phase asthmatic response. These findings represent the differential effect of the PDE4 inhibitor on airway hyperresponsiveness and the effect on key inflammatory cells and mediators involving in the early-phase and late-phase asthmatic response. Since both EAR and LAR are considered the distinct processes involving asthma pathophysiology from the different mediators, for instance, IgE, cysteinyl leukotriene, and histamine, they play their roles, together with inflammatory cells recruitment eosinophils and neutrophils (O’Byrne, 1998).

Apart from oral PDE4 inhibitor roflumilast, CH6001, a novel inhaled PDE4 inhibitor, has shown late-phase asthmatic reaction attenuation and sputum eosinophils reduction in allergic asthma compared to placebo (Singh et al., 2016a). Additionally, allergen-induced late-phase asthmatic responses were attenuated by inhaled GSK256066, a selective PDE4 inhibitor, in steroid-naïve allergic asthma patients compared to placebo (Singh et al., 2010). Moreover, the novel oral PDE4 inhibitor MEM1414 has been tested for its effect on allergen-induced asthmatic response in steroid-naïve allergic asthma. MEM1414 abrogated LAR without an effect on early asthmatic response. Also, the *ex vivo* experiment has shown that ME1414 reduced lipopolysaccharide (LPS)-stimulated TNF-a release as well as LTB4 from the whole blood of asthmatic patients (Leaker et al., 2014). These attenuation effects of a PDE4 inhibitor on neutrophilic inflammation were tested later for asthma. Since neutrophilic inflammation may be key for severe steroid-resistant asthma and exercise-induced asthma, exercise-induced asthma can be modulated by roflumilast and was confirmed in the *ex vivo* model using LPS stimulating TNF release (Timmer et al., 2002). A summary of the effects of roflumilast and other oral or inhaled PDE4 inhibitors is shown in Table 2.

**TABLE 2:** Effects of roflumilast and the novel PDE4 inhibitor on allergen-induced early and late asthmatic response and airway inflammation in asthmatic patients.

Author, year	Patient characteristic	Interventions	Duration (weeks)	Outcomes
Louw et al. (2007)	Mild allergic asthma	Single-dose RF 1000 µg vs. PBO	Single dose	Preventing late-phase asthmatic response to allergen (2–9 h after challenge). No effect on early-phase asthmatic response
Van Schalkwyk et al. (2005)	Mild-to-moderate allergic asthma	RF 250 µg and RF 500 µg OD	1 week	Preventing early- and late-phase asthmatic response to allergen (dose-dependent)
Gauvreau et al. (2011)	Mild allergic asthma	RF 500 µg OD vs. PBO	2 weeks	Prevention of late-phase asthmatic response to allergen. Reduction of sputum eosinophils and neutrophils. No effect on early-phase asthmatic response
Singh et al. (2016a)	Atopic asthma, ICS-naïve	Inhaled CHF6001 400 µg/1200 µg or PBO OD via DPI	1 week	Prevention of late-phase asthmatic response to allergen. Nonsignificant reduction of sputum eosinophils
Singh et al. (2010)	Atopic asthma, ICS-naïve	Inhaled GSK256066 87.5 µg vs. PBO OD	1 week	Prevention of late-phase asthmatic response to allergen
Leaker et al. (2014)	Atopic asthma, ICS-naïve	Oral MEM 1414 600 mg BID vs. PBO	2 weeks	Prevention of late-phase asthmatic response to allergen. No effect on early-phase asthmatic response. Reduction of LPS-stimulated TNF-α and LTB4 release from whole blood assay

ICS, inhaled corticosteroid; RF, roflumilast; PBO, placebo; DPI, dry-powder inhaler; OD, once daily; LPS, lipopolysaccharide; TNF-α, tumor necrosis factor-alpha; LTB4, leukotriene B4.

Roflumilast for treating asthma has been tested in clinical trials for 2 decades. However, this drug has never been approved for asthma. The different doses of roflumilast improved lung function in patients with asthma in a dose-dependent fashion. The efficacy of the drug for improving airway function in asthma is comparable to low-dose inhaled beclomethasone (Bousquet et al., 2006). Despite the fact that forced expiratory volume in 1 s (FEV_1_) is a reproducible and reliable physiologic surrogate for testing asthma drugs, roflumilast also improves other clinically relevant physiologic parameters, for instance, peak expiratory flow rate (PEFR) representing large airway function. The FVC improvement in roflumilast represents the reduction of the degree of air trapping as the effect of an orally ingested drug on small airways (Bousquet et al., 2006). By comparing with other pivotal asthma maintenance treatments, roflumilast improves lung function (FEV_1_) and asthma symptoms in mild-to-moderate asthmatic patients compared with both low-dose beclomethasone and montelukast (Bateman et al., 2015). Pooled data of randomized clinical studies in adults with asthmatic age ranging from 18 to70 years have shown that orally administered roflumilast in different doses improves asthma outcome in terms of modulating airway inflammation measured by sputum eosinophils, neutrophils, and exhaled nitric oxide, attenuating allergen-induced allergic asthma response and increasing lung function surrogates (Bardin et al., 2015). However, in contrast to COPD, the lack of adverse effects on body weight loss has been addressed. Hence, roflumilast deserves to be an alternative treatment to ICS in patients with mild-to-moderate asthma.

Despite the fact that ICS is a mainstay treatment of asthma, the effect of high dose corticosteroid on airway caliber is a flat dose response. The lack of benefit after increasing the ICS dose results in a substantial increase in adverse effects (Lipworth, 1996). For these reasons, adding on drugs, for instance, the long-acting β2 agonist or montelukast, is preferred to increasing the dose of ICS (Currie et al., 2005). The added benefit of LABA to ICS is better than add-on montelukast which has been shown in meta-analysis in short-term airway function improvement (Ram et al., 2005; Ducharme et al., 2006). ICS/LABA is standard for escalating treatment persistent asthma already on ICS. The available oral asthmatic controllers, for instance, montelukast and sustained-release theophylline, are added on to ICS/LABA therapy for improving asthma control, which has been examined. Hence, in terms of being added on the agent for uncontrolled asthma despite ICS/LABA combination, add-on montelukast to ICS/LABA improves asthma control in a short-term open-label study (Virchow et al., 2010). The short-term study is evaluating the effect of adding on 500 mcg roflumilast and 10 mg montelukast compared to montelukast alone for adding on ICS/LABA in patients with asthma. Combining roflumilast and montelukast has been shown to improve FEV_1_ compared to montelukast alone for moderate-to-severe asthmatic patients who were already on ICS/LABA (Bateman et al., 2016). The improved lung function for both FEV_1_ and FVC has shown the additional benefit of roflumilast to backbone asthmatic treatment. The potential mechanisms explain the additive benefit of roflumilast coadministered with ICS/LABA which is due to attenuation of airway inflammation for the ICS or synergistic bronchodilator effect with LABA. The interplay between the glucocorticoid receptor and cAMP signaling pathways may contribute to the additional effect. This mechanism explains how LABAs and PDE4 inhibitors enhance the clinical efficacy of glucocorticoids in inflammatory lung diseases (Giembycz and Newton, 2015). Also, the ovalbumin-asthmatic mice model has shown that coinhalation of roflumilast and fluticasone significantly decreased airway hyperresponsiveness and improved inflammation in bronchoalveolar lavage fluid and pathological changes compared to coinhalation of formoterol and fluticasone (Murad et al., 2017). Orally administered roflumilast in ovalbumin-induced asthmatic mice has been shown to attenuate goblet cell hyperplasia and pulmonary fibrosis and airway remodeling via inhibiting stem cell factor (SCF)-induced cell proliferation of fibroblasts (Kim et al., 2016). The benefit of roflumilast for improving airway inflammation, decreasing AHR, and increasing lung function is demonstrated. Nevertheless, the benefit of improving asthma control and preventing future risk including lung function decline due to remodeling is also the opportunity for novel asthma drug development. Interestingly, the effect of add-on roflumilast to ICS or ICS/LABA has been shown in terms of lung function in clinical studies for severe uncontrolled asthma. Lastly, the effect on clinical asthma control and asthma-related future risk including exacerbation and airway remodeling still need to be investigated in the long-term clinical studies. A summary of the clinical efficacy of roflumilast on asthma patients in clinical trials is shown in Table 3.

**TABLE 3:** Clinical efficacy of roflumilast on asthma patients in clinical trials.

Author, year	Patient characteristic	Interventions	Duration (weeks)	Outcomes
Bousquet et al. (2006)	Mild asthma, FEV_1_ 50–85%	RF 5000 µg OD vs. BDP 200 µG BID	12 weeks	Comparable effect of RF and BDP for improving FEV_1_ and FVC. Improved asthma symptom scores and reduced rescue medication use from baseline
Timmer et al. (2002)	Exercise-induced asthma	RF 500 µg vs. PBO OD	4 weeks	RF prevents FEV_1_ fall after exercise challenge in days 1 and 14 compared to PBO. Reduced LPS-stimulated TNF-α
Bateman et al. (2016)	Moderate-to-severe asthma that remained uncontrolled asthma despite ICS/LABA	RF 500 µg plus MT 10 mg vs. PBO plus MT 10 mg (cross-over)	4 weeks	RF plus MT increased FEV_1_ and FVC compared to PBO plus MT. RF plus MT improved ACQ-5 and ACQ-7 from baseline but not different from PBO plus MT. Reduced urinary LTE_4_

ICS, inhaled corticosteroid; RF, roflumilast; PBO, placebo; BDP, beclomethasone dipropionate; MT, montelukast; ICS/LABA, inhaled corticosteroid/long-acting β2 agonist; OD, once daily; BID, twice a daily; ACQ, asthma control questionnaire (ACQ); LPS, lipopolysaccharide; TNF-α, tumor necrosis factor-alpha; LTE_4_, leukotriene E4.

The reason underlying the paucity of clinical studies regarding PDE4 inhibitors in asthma is the comparable efficacy of PDE4 inhibitors to ICS therapy. In addition, prominent side effects of roflumilast, the selective PDE4 inhibitor, warrant the clinical utility in asthma. One way is the ICS that is the major or cornerstone treatment of asthma. Despite this, the oral leukotriene modifier is comparable to ICS in asthma treatment (McIvor, et al., 2009). In addition, asthma with coexisting allergic rhinitis may benefit from adding montelukast to ICS or ICS/LABA (Virchow et al., 2010). Since the addition of LABA to ICS provides a better benefit for lung function improvement compared to montelukast (Ducharme et al., 2006), the synergistic effects of ICS and LABA have been widely accepted (Johnson, 2002). The paucity of evidence regarding the use of added PDE4 inhibitors to ICS is a major consideration. In contrast to COPD, adding roflumilast to LABA or LAMA in COPD patients has shown the benefit of improving lung function (FEV_1_). Moreover, adding roflumilast to ICS/LABA or ICS/LABA/LAMA has shown the benefit of COPD exacerbation reduction as mentioned previously. The combined oral PDE4 inhibitor including roflumilast to ICS needs more studies examining the synergistic effect of PDE4 inhibitor and ICS on asthma and clinical efficacy.

**FIGURE 2:**
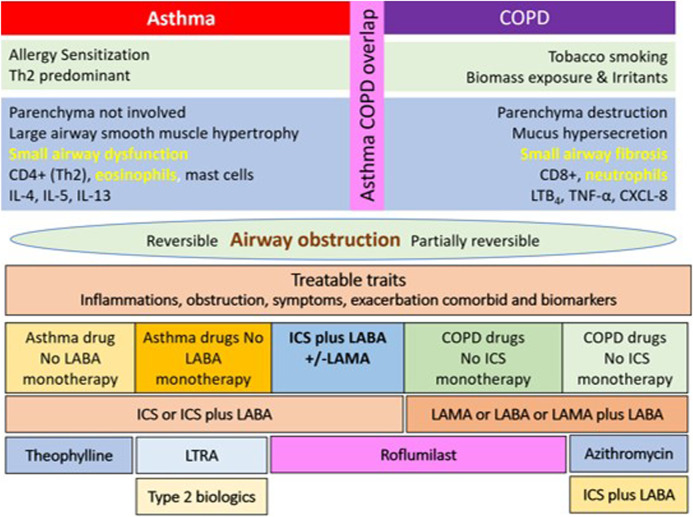
The diagram of the possible role and position of roflumilast for being asthmatic controller in a stepwise approach to asthma pharmacologic treatment. ICS: inhaled corticosteroid; SMI: soft mist inhaler; OCS: oral corticosteroid.

Roflumilast has potential roles for being an asthmatic controller as mentioned previously. The position of the drug in add-on therapy to ICS or ICS-containing regimens is shown in Figure 2. Apart from PDE4 inhibitors, PDE1 and PDE3 are targets for novel antiasthmatic treatments. The molecular mechanisms underlying the effect of the PDE3 inhibitor on asthma and obstructive airway disease are that PDE3 is expressed in both structural cells and immune cells. Structural cells of lungs include smooth muscle cells, epithelial cells, and endothelial cells. The PDE3 regulates immune cells, including dendritic cells, monocytes, B-cells, NK cells γδT cells, αβT-cells, T-cells, macrophages, eosinophils, and neutrophils (Beute et al., 2018). Further clinical studies examining the effect of PDE1 and PDE3 inhibitors on asthma are required in drug development processes.

## PDE4 Inhibitor in Asthma COPD Overlap

There is an interest in the characteristics or clinical traits of patients with the diagnosis of both asthma and COPD. Due to the lack of a well-accepted and precise definition of ACO, it is problematic to define the population in clinical trials of ACO. Hence, the treatments of ACO are extrapolated from asthma and COPD studies. COPD patients presenting with blood or systemic eosinophilia have demonstrated the clinical response to ICS for the reduction in an exacerbation. Also, management of severe asthma patients requires phenotypic treatment and monoclonal antibodies (Maselli et al., 2019). Roflumilast reduces sputum inflammatory cells in COPD patients. This can postulate the benefit of adding oral roflumilast to asthma and COPD. The potential mechanisms of roflumilast on asthma, COPD, and ACO include anti-inflammatory, bronchodilator, enhancing mucociliary clearance, modulating airway hyperresponsiveness, preventing airway remodeling, and retarding airway fibrosis (Zhang et al., 2018).

**FIGURE 3:**
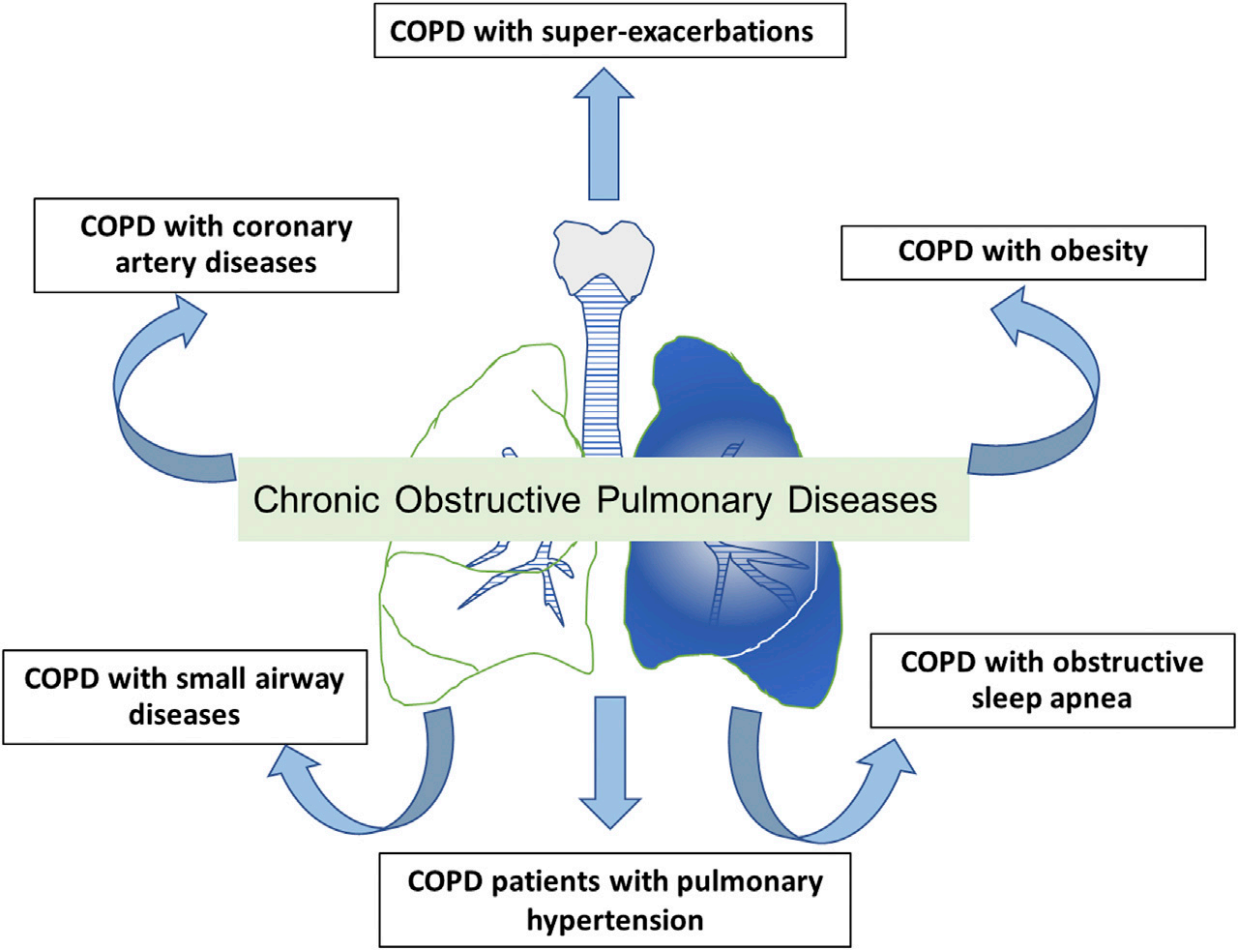
The potential role and positions of roflumilast for being maintenance therapy in patients with asthma-COPD overlap (ACO). ICS: inhaled corticosteroid; LAMA: long-acting muscarinic antagonist; LABA: long-acting β_2_ agonist.

The modulation of inflammatory cytokines IL-6, IL-8, IL-17, and TNF-α is key to the anti-inflammatory properties of roflumilast in both asthma and COPD. However, the improvement of the ventilation defect by increasing FEV_1_ to LAMA or ICS/LABA in COPD and increasing FEV_1_ in asthma has been shown in clinical trials. Also, the improvement of FEV_1_ and the modulation of AHR have been shown in roflumilast-treated patients with asthma. To date, there is no definite evidence of the clinical benefit of roflumilast in ACO patients. In a bronchial biopsy study that examined the anti-inflammatory effects of roflumilast on patients with COPD, in comparison with placebo, roflumilast was associated with a significant reduction in eosinophils in the bronchial biopsy and significant reductions in both absolute and differential eosinophil cell counts in induced sputum. However, peripheral blood eosinophil counts were not affected (Rabe et al., 2018). These findings elucidate the mechanism of cough in the presence of eosinophilic airway inflammation and the effect of short-term oral PDE4 inhibitor therapy such as roflumilast. The potential role of roflumilast in ACO is shown in Figure 3.

## PDE4 Inhibitors in Bronchiectasis

**FIGURE 4:**
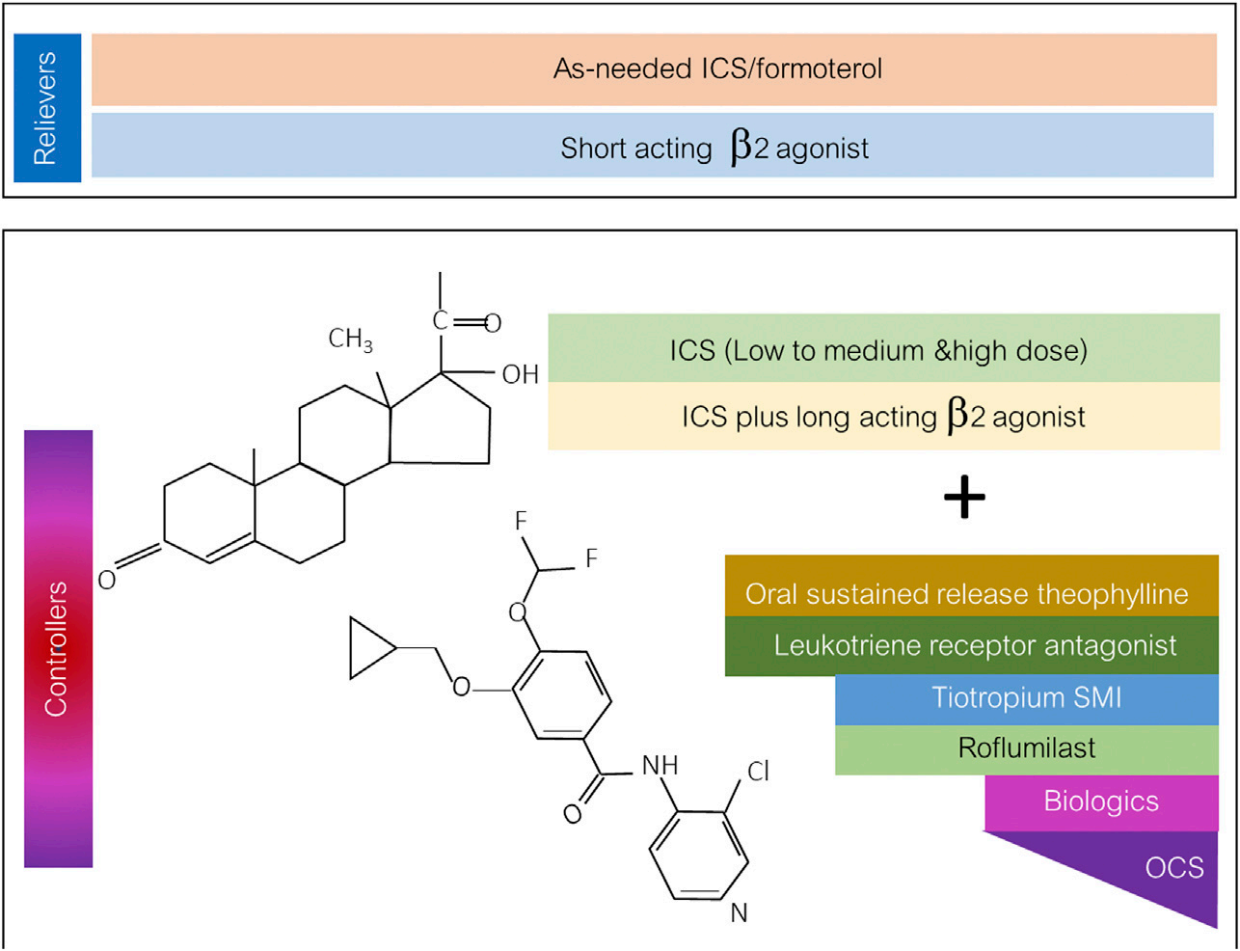
Mechanisms of roflumilast in non-CF bronchiectasis for both modulating inflammatory cells and enhancing structural cell function of bronchiectasis.

Bronchiectasis is a chronic suppurative airway condition presenting with cough and phlegm production. The diagnosis of bronchiectasis requires the persistent dilatation of the bronchi. The pathogenesis of diseases is a vicious cycle of chronic airway inflammation, colonization of bacteria, and permanent dilatation of the bronchi causing repeated infection and inflammation of the airway (Cole, 1986). The current treatment of bronchiectasis from European Respiratory Society (ERS) guidelines includes eradiation of bacterial colonization in particular *P. aeruginosa*, long-term antibiotic therapy for repeated infected bronchiectasis for more than 3 times a year, pulmonary rehabilitation, bronchial hygiene therapy, and a bronchodilator for patients with breathlessness symptoms. The benefit of ICS in bronchiectasis is minimal and should not be recommended for the treatment of bronchiectasis in the absence of ICS indication such as asthma. For these reasons, the roles of novel anti-inflammatory therapies have been extensively studied in clinical trials. The anti-inflammatory therapy and immune-modulatory drugs, for instance, NE inhibitor, GM-CSF inhibitor, and selective CXCR2 antagonist, which modulate neutrophil function have been tested for their safety in short-term studies. However, the results of the clinical outcome of bronchiectasis are lacking (Chalmers and Chotirmall, 2018). Potential mechanisms of roflumilast for treating bronchiectasis may be modulating neutrophil function, improving mucous and ciliary function, and the bronchodilator effect. To date, there are no current clinical studies of roflumilast’s effect on the clinical outcome of patients with bronchiectasis. The current clinical trials of roflumilast for noncystic bronchiectasis which are under process are shown (Park, 2014) in Table 4. The potential mechanisms of roflumilast for non-CF bronchiectasis are shown in Figure 4.

**TABLE 4:** Clinical efficacy of roflumilast on noncystic fibrosis bronchiectasis patients in clinical trials.

NTC number or investigator	Participants	Intervention	Comparators	Outcome
NCT03988816	Age ≥18 years, CT diagnosed bronchiectasis, and FEV_1_ <60% predicted. Chronic bronchitis, ≥ 2 infectious exacerbations in the last year	Roflumilast 500 µg OD 12 weeks	Oral placebo	Quality of life (SGRQ), lung function, and mucus properties
NCT03428334	Aged ≥18 years, HRCT confirmed bronchiectasis and sputum production (≥10°ml per day)	Roflumilast 500 µg OD 4 weeks	Oral placebo	Light microscopy and hemocytometry for sputum leukocyte density
J Park et al. NTC 015801748	Symptomatic non-CF bronchiectasis	Roflumilast 500 µg OD	Oral placebo	COPD assessment test (CAT score) and SGRQ-C score
		Roflumilast 250 µg OD 16 weeks		

## PDE4 Inhibitors in Chronic Cough

The cough symptom is common in chronic respiratory diseases including asthma and COPD. The previous section examined the benefit of roflumilast on cough and sputum production in patients with COPD. Roflumilast attenuates cough and sputum in severe COPD patients who are already treated with inhaled combination therapy. There is the improvement of Evaluation of the EXAcerbation of Chronic Pulmonary Disease Tool–Patient Reported Outcomes (EXACT-PRO) score after 52 weeks of roflumilast treatment (Sethi et al., 2017). However, in addition to the benefit of roflumilast on cough symptoms in COPD patients with chronic bronchitis, the potential mechanisms are the decrease in MUC5AC expression in bronchial epithelium, increases in CFTR dynamic activation of airway epithelium cells, and enhanced ciliary beat of smoke-injured human bronchi (Liu et al., 2005; Mata et al., 2005; Milara et al., 2012). Chronic cough of various etiologies is reported as a troublesome complaint of patients in clinical practice. Despite its high burden, the therapeutic strategies of both pharmacologic and nonpharmacologic choices are limited. The lack of effective antitussive and the lack of mechanistic research to elucidate the mechanisms of cough are key issues. One of these mechanisms and therapeutic targets is the transient receptor potential (TRP) ion channels, receptors activated by such chemical stimuli, temperature, osmotic stress, and mechanical stress (Grace et al., 2013). Since cough reflex sensitivity via TRP channels, for instance, transient receptor potential vanilloid-1 (TRPV1) and transient receptor potential ankyrin-1 (TRPA1), is associated with chronic cough from several diseases and disorders, pharmacologic treatments have been purposed by the modulating of TRP. Hence, this novel mechanism has potential for the treatment of chronic cough-related airway diseases including TRP inhibitor and roflumilast (Bonvini and Belvisi, 2017). The inhibition of PDEs results in bronchodilation, suppression of TRPV channels, and anti-inflammatory action in cough suppression (Mokry et al., 2018). To date, there are no clinical studies that have examined the benefit of roflumilast, its mechanism on TRP channels, and its clinical efficacy on chronic troublesome cough (Preti et al., 2012).

## Discussion

Roflumilast and other selective PDE4 inhibitors exhibit definite therapeutic roles in patients with COPD and possible roles in other chronic airway diseases. The mechanisms of roflumilast and drugs in this class are the modulation of the airway and systemic inflammation. However, the heterogeneity of airway inflammation including eosinophilic and neutrophilic is a treatment target for these anti-inflammatory drugs. They modulate airway inflammation in both asthma and COPD and improve airway function in both bronchodilator and nonbronchodilator effects. To date, roflumilast is the only PDE4 inhibitor approved for the treatment of COPD with chronic bronchitis phenotypes in patients who suffer from exacerbations. However, its role is limited by being added to backbone COPD treatment in terms of long-acting bronchodilators with or without ICS. The possible therapeutic role in severe asthma and ACO is promising but a large-scale study to support clinical use is needed. Furthermore, roflumilast has a clinical effect on extrapulmonary manifestation including metabolic derangement in COPD patients by reducing fat mass and glycemic control. The therapeutic opportunity in chronic airway inflammatory airway diseases including noncystic bronchiectasis and modulation of chronic persistent cough is promising and under clinical investigation. To sum up, roflumilast and PDE4 inhibitors may be classified as broad-spectrum oral anti-inflammatory treatment of chronic airway diseases. Preclinical studies elucidating the mechanisms of action are important as are the well-designed clinical studies examining the clinical efficacy of these drugs in chronic airway diseases other than COPD.
